# Responding to the syphilis outbreak in Japan: piloting a questionnaire to evaluate potential risk factors for incident syphilis infection among men who have sex with men in Tokyo, Japan, 2015

**DOI:** 10.5365/WPSAR.2016.7.2.001

**Published:** 2016-07-11

**Authors:** Masahiro Ishikane, Yuzo Arima, Ichiro Itoda, Takuri Takahashi, Takuya Yamagishi, Tamano Matsui, Tomimasa Sunagawa, Kazunori Oishi, Makoto Ohnishi

**Affiliations:** a Field Epidemiology Training Program, National Institute of Infectious Diseases, Tokyo, Japan.; b Division of Global infectious diseases, Department of Infection and Epidemiology, Graduate School of Medicine, Tohoku University, Miyagi, Japan.; c Disease Control and Prevention Center, National Center for Global Health and Medicine, Toyama, Shinjuku-ku, Tokyo, Japan.; d Infectious Disease Surveillance Center, National Institute of Infectious Diseases, Tokyo, Japan.; e Shirakaba Clinic, Tokyo, Japan.; f Department of Bacteriology I, National Institute of Infectious Diseases, Tokyo, Japan.

In Japan, notifications of syphilis reported through the national surveillance system have been increasing recently (*1*, *2*) similar to many other developed countries. (*3*, *4*) During 2013–2014, this increase was associated with men who have sex with men (MSM). (*1*, *2*) In response to this increase, the Ministry of Health, Labour and Welfare of Japan has been raising awareness and disseminating prevention messages. Recent reports indicated that factors associated with syphilis among MSM were low educational attainment, sex with casual partners without a condom and coinfection with other sexually transmitted infections (STIs). (*1*, *2*) Using mobile phone applications and the Internet to seek sex partners has also been reported as potential risk factors for STIs, including syphilis, among MSM. (*5*, *6*) There has been no study regarding potential predictors for syphilis acquisition in Japan. To investigate the possible reasons for the syphilis outbreak among MSM in Tokyo, a case-control study based on a questionnaire assessing potential risk factors for incident syphilis was proposed. To pretest the tool, we piloted the questionnaire and report key findings. We remind those in public health practice of the importance of pretesting questionnaire tools whenever possible.

The pilot was conducted at the same clinic proposed for the case-control study, located in central Tokyo, the epicentre of the syphilis outbreak in Japan. The clinic serves sexual minority populations, and the majority of the patients are MSM from the metropolitan Tokyo area. This clinic serves about 500 HIV-positive patients annually. It also reported the largest number of syphilis cases from a single facility in Tokyo in 2013 (*n* = 76, 18% of cases from Tokyo). (*7*)

With or without acquisition of syphilis, the first six MSM who attended the clinic for any reason on the afternoon of Wednesday, 7 January 2015, were invited to participate. The questionnaire was developed based on those from recent studies (*8*, *9*) and adapted to the Japanese context. The questions were about socio-demographics; health conditions, including a past STI history; sexual activities, including partner characteristics; and an open-ended question for comments and suggestions to improve the tool. The self-administered questionnaire was completed by the respondent in a private location within the facility. Participants were asked if they had ever had sex with men, and the information was verified by chart review. The institutional ethics committee of the National Institute of Infectious Diseases in Japan reviewed and approved the study protocol.

All (6/6) invitees agreed to participate in the study. All questions were responded to with no missing data. The median age was 38 (range 27–48) years; all had their sexual debut with another male by 20 years of age. History of STIs other than HIV or syphilis (e.g. anogential human papillomavirus [HPV] infections) was common (**Table 1**). Three reported sexual activity in the past six months, and included both insertive and receptive anal and oral sex. Methods for seeking partners were diverse, including the Internet, mobile phone applications, cruising spots and public baths. Among the recently sexually active participants, one with a history of syphilis reported using a condom “occasionally,” while the other two reported using it every time. One participant (participant 3), who was the most sexually active with eight casual partners, sought partners via multiple approaches and reported use of a sex toy and alcohol intake during sex; however, he reported condom use “every time.”

**Table 1:** Characteristics of six MSM who visited STI clinic in central Tokyo, Japan, 2015

	Participants					
	1	2	3	4	5	6
**Socio-demographic characteristics**						
Age (years)	29	32	27	43	45	48
Highest level of education	University degree	High school diploma	University degrees	University degree	University degree	High school diploma
Employment status	Part time	Student	Student	Full time	Full time	Full time
Age of first sex with a male (years)	17	16	15	20	13	15
**STI status**						
Syphilis status	Negative	Past infection	Negative	Negative	Negative	Negative
HIV infection status*	Positive	Positive	Negative	Negative	Positive	Positive
Past STIs other than syphilis and HIV	None	Hepatitis B, Anogential HPV	Chlamydia	Anogential HPV	Anogential HPV	Hepatitis B
**Sexual activity in the past six months**						
Number of partners	1	4	8	None	None	None
Partner type	Steady	All casual	All casual	–	–	–
Method of seeking sexual partners	Internet	Internet	Mobile phone applications; cruising spot; public bath	–	–	–
Average frequency of sex (anal or oral)	< 1/month	≥ 1/month and < 1/week	1/week	–	–	–
Anal sex type	Insertive, Receptive	Insertive, Receptive	Insertive, Receptive	–	–	–
Oral sex type	Insertive, Receptive	Insertive, Receptive	Insertive, Receptive	–	–	–
Condom used during anal sex	Every time	Occasionally	Every time	–	–	–
Condom used during oral sex	Every time	Occasionally	Every time	–	–	–
Alcohol intake during sex	None	None	3–5 servings of beer	–	–	–
Recreational drug use during sex	None	None	None	–	–	–
Sex toy use during sex	None	Anal toy	Anal, Oral toy	–	–	–

*Median CD4 count of the four HIV-positive participants was 416 (range 284–1018) /μl and HIV-RNA was well suppressed (all were receiving antiretroviral therapy at the time of study).

AIDS, acquired immunodeficiency syndrome; HIV, human immunodeficiency virus; HPV, human papillomavirus; MSM, men who have sex with men; and STI, sexual transmitted infection.

Within this small pilot sample, important themes emerged. First, large variations in responses were obtained, and we found that the participants used a variety of methods to seek sex partners. While temporally correlated, the recent rise in syphilis may be more complex than a singular attribution to the increase in partner-finding mobile phone applications as recently reported among MSM in Asia. (*6*) Such findings underscored the rationale for a thoughtfully designed questionnaire to address this concern along with other potential risk factors. Notably, anogential HPV prevalence was high in our sample, which has also been reported recently among MSM in Japan, (*10*) so we added “anogential HPV” as one of the choices under the STI history section in the revised questionnaire.

Our experience iterated the importance of pretesting tools that deal with sensitive topics among hard-to-reach populations. One participant commented that the terminology used for partner-seeking methods could be improved to better reflect the language used among the target population; thus, for the question regarding methods of seeking sexual partners, three distinct categories were collapsed into one “cruising spots (outdoors/playing rooms)” in the revised questionnaire. Such modifications are important outcomes of pretesting to improve the validity of measurements, especially with marginalized groups. Better understanding of the target group’s language can also help in risk communication.

This study had several limitations. First, the sample size was small. Nevertheless, this pilot achieved its aim to pretest the questionnaire to assess for usability, including qualitative feedback and to capture key themes. Second, while convenience sampling was used for practical reasons, we attempted to minimize selection bias by choosing a regular weekday afternoon and invited the first six patients who attended the clinic to participate. However, in our sample, HIV prevalence was higher (4/6) than that of the overall MSM clinic patient population (37% during 2007–2011 [Itoda I, Shirabaka Clinic, unpublished data, 2011]). As HIV-positive patients visit the clinic frequently for routine monitoring, their chance of being selected could have been high. Stratified or purposive sampling to select MSM by HIV seropositivity could have been useful to approach certain themes by HIV status. A focus group session may also have captured additional important thematic elements. While none of the six had incident syphilis, this should not affect our objective to pretest the tool as controls are also needed for a case-control study.

Our pilot study indicated that there are currently multiple avenues of seeking sex partners, highlighting the need for a careful evaluation of risk factors for syphilis acquisition. Our experience also iterated the importance of pretesting to better reflect the actual language used among the target demographic. As questionnaires are commonly used to test hypotheses and target interventions in outbreak responses, pretesting should be considered an essential part of the response whenever feasible. This process also helps us better understand relevant social issues and potential entry points for control. Currently, the revised questionnaire is being implemented in the case-control study among MSM in Tokyo, and we hope that the findings will lead to evidence-based prevention messages and interventions.
